# Wheat grain moxibustion ameliorates oxidative stress in PTU-induced hypothyroid rats via cAMP/PKA pathway activation

**DOI:** 10.3389/fendo.2025.1612008

**Published:** 2025-10-22

**Authors:** Jing Qin, Tingting Liu, Xiangyue Zhang, Xueni Chen, Jie Qin, Muhammad Shahzad Aslam, Huimin Feng, Yanhui Zhang, Xiaoxu Wang, Tiansheng Zhang, Chongyao Hao

**Affiliations:** ^1^ Shanxi University of Traditional Chinese Medicine, Jinzhong, China; ^2^ School of Traditional Chinese Medicine, Xiamen University Malaysia, Sepang, Selangor, Malaysia; ^3^ School of Medicine, Department of Traditional Chinese Medicine, Xiamen University, Fujian, China; ^4^ Shanxi Acupuncture and Moxibustion Hospital, Taiyuan, Shanxi, China

**Keywords:** wheat grain moxibustion, hypothyroidism, oxidative stress, cAMP/PKA pathway, PDE3A, CREB

## Abstract

**Background:**

Hypothyroidism is a systemic hypometabolic disorder characterized by thyroid hormone deficiency and is closely associated with oxidative stress. The cAMP/PKA signaling pathway is critically involved in regulating thyroid hormone secretion and reducing oxidative stress. This study investigates whether wheat grain moxibustion alleviates hypothyroidism in a hypothyroidism model by attenuating oxidative stress and activating the cAMP/PKA signaling pathway.

**Methods:**

The hypothyroid rat model was established by administering propylthiouracil. Following wheat grain moxibustion treatment, rat general condition, behavioral performance, and thyroid function were assessed. Then thyroid tissues and serum samples were collected. Thyroid tissue microstructure was examined by hematoxylin-eosin staining. Serum levels of triiodothyronine, thyroxine, thyroid-stimulating hormone, free triiodothyronine, free thyroxine, reactive oxygen species, malondialdehyde, catalase, superoxide dismutase, glutathione peroxidase, and cAMP were quantified using ELISA. Protein expression of signaling pathway components was detected via WB, while corresponding mRNA levels were measured by RT-PCR. To clarify the relationship between cAMP/PKA signaling and oxidative stress, the PKA inhibitor H-89 was administered to a subset of wheat grain moxibustion-treated rats.

**Results:**

Wheat grain moxibustion treatment ameliorated the general condition, behavioral performance, and thyroid hormone profiles in propylthiouracil-induced hypothyroid rats. Furthermore, it significantly reduced serum levels of reactive oxygen species and malondialdehyde, while enhancing the activities of catalase, superoxide dismutase, and glutathione peroxidase, collectively indicating attenuated oxidative stress. Concurrently, wheat grain moxibustion activated the cAMP/PKA pathway, demonstrated by elevated serum cAMP levels, downregulated phosphodiesterase 3A expression, upregulated PKA expression, and increased phosphorylation of CREB in thyroid tissues. Notably, pharmacological inhibition of this pathway with H-89 reversed the ameliorative effects of wheat grain moxibustion on oxidative stress.

**Conclusion:**

Wheat grain moxibustion ameliorates oxidative stress in a propylthiouracil-induced hypothyroid rat model, mediated through activation of the cAMP/PKA signaling pathway. These results indicate its potential therapeutic utility in hypothyroidism management and mitigation of oxidative injury.

## 1 Introduction

Hypothyroidism (HT), a systemic hypometabolic disorder caused by a deficiency of thyroid hormones, is a common endocrine disorder in women and older adults. There are four types of HT, of which primary hypothyroidism is the most common (95%-99%) (1). The most noteworthy aspect of diagnosing HT is high levels of thyroid stimulating hormone (TSH) with low levels of free thyroxine (FT4) (2). The clinical features of HT are complex and not specific, making it difficult to diagnose, but it can adversely affect the patient’s health or even cause death. Oral levothyroxine (LT4) remains the primary treatment for HT in clinical practice (3, 4). However, its therapeutic efficacy is often compromised by challenges such as over- or under-dosing, leading to suboptimal control of biochemical parameters (5). Additionally, long-term adherence is frequently poor due to the requirement for prolonged administration.

Oxidative stress (OS) refers to a pathophysiological state characterized by an imbalance between the generation of pro-oxidants and the capacity of antioxidant defense mechanisms (6). This disruption leads to molecular and cellular alterations, resulting in potential damage to lipids, proteins, and DNA. The thyroid gland exhibits high metabolic activity in both generating and eliminating reactive oxygen species (ROS), which are key markers of OS. In the context of thyroid dysfunction, ROS production is significantly elevated (7) which excess ROS overwhelms the endogenous antioxidant defense mechanisms, resulting in cellular damage, apoptosis, and ultimately cell death. These processes contribute critically to the pathogenesis of HT (8). ROS are highly reactive, attacking nearby polyunsaturated fatty acids to form malondialdehyde (MDA) (9), which can indirectly reflect the level of free radicals in the body and directly correlate with the degree of cellular damage. The first line of defence mechanism against OS is the most effective and involves antioxidant enzymes such as catalase (CAT), superoxide dismutase (SOD), and glutathione peroxidase (GPx). This line of defence plays an irreplaceable role in the disproportionation of superoxide radicals and hydrogen peroxide (H_2_O_2_) (10). The specific manifestation of OS in HT is an increase in oxidative levels and a decrease in antioxidant levels.

Cyclic adenosine monophosphate (cAMP) was the first-second messenger described and the most widely used. CAMP is produced from adenosine triphosphate by adenylate cyclase, and its hydrolytic degradation is catalysed by phosphodiesterase (PDE) (11); in addition, cAMP-dependent protein kinase (PKA), a downstream molecule, promotes the phosphorylation of cAMP-responsive element-binding proteins (CREBs), which ediate gene transcription, cell migration, mitochondrial homeostasis, cell proliferation, and cell death (12), this pathway is indispensable in the maintenance and dysregulation of energy homeostasis (13). It has been shown that copper(II) treatment induces OS and inflammasome-mediated pyroptosis that may be associated with copper(II)-induced impairment of postsynaptic cAMP, PKA, and CREB signalling (14); cAMP/PKA signalling pathway can attenuate H_2_O_2_-induced oxidative damage to GCs through the mitochondrial pathway by participating in the process of enhancing antioxidant capacity, restoring mitochondrial function and inhibiting apoptosis (15), which all suggest that the cAMP signalling pathway plays a huge role in the regulation of OS.

Moxibustion therapy has been a Chinese method of treating diseases since ancient times and is still clinically useful. Wheat grain moxibustion (WGM) is a method of moxibustion in which moxa is rolled or made into wheat-sized moxibustion grains using molds and placed on acupoints to ignite them, which has the advantages of high stimulation volume, ease of use, and low cost. Some studies have shown that WGM can up-regulate SOD activity, down-regulate MDA content, and inhibit cellular OS damage (16). In addition, moxibustion therapy positively regulates the activation or inhibition of the cAMP signalling pathway (17–19). Our group’s preliminary study had the following findings, the first of which is that WGM can affect the levels of TSH, FT3, FT4, TT4, TPOAb, TGAb and NIS, suggesting that WGM improves the function of the thyroid gland (20, 21). The second is that WGM affects the levels of cytokines such as IFN-γ, TGF-β1, IL-4, IL-10, IL-23, and other cytokine levels, suggesting that maitake moxibustion modulates the immune dysregulation present in HT (22, 23). The third one is that WGM can affect MR, GR, and acetylcholine content in hippocampal tissues, suggesting that WGM improves the depressive state, learning and memory ability in HT (24, 25). During the study, it was also found that the developmental process of HT and the mechanism of action of WGM in the treatment of HT, cAMP, and PKA (21) play an important role but have not been studied in depth before.

This study employed a PTU-induced rat model of HT to investigate whether WGM ameliorates OS in HT rats and to examine the potential involvement of the cAMP/PKA signaling pathway in this process.

## 2 Materials and methods

### 2.1 Animal grouping and model preparation

Sixty 8-week-old specific pathogen-free Sprague-Dawley (SD) rats (30 males and 30 females), each weighing 200 ± 20 g, were obtained from Spivey (Beijing) Biotechnology Co., Ltd. [Production Licence No. SCXK (Beijing) 2019-0010]. All animals were housed in an SPF-grade facility under standard conditions: a 12-h light/12-h dark cycle at 22–25°C with a daily temperature fluctuation of no more than 3°C, 45–50% relative humidity, and free access to chow and water. After one week of acclimation, the rats were randomly divided into two groups: a normal group (NG; n =10; 5 males and 5 females) and a modeling group (n = 50). The 50 rats in the modeling group were gavaged once daily with 0.1% propylthiouracil (PTU, batch no. 2307N12, Shanghai Chaohui Pharmaceutical) at 1 mL per 100 g of body weight for 4 weeks to establish the HT model. After examining the success of modelling, thirty were selected by random number method and then was divided into model group (Model), levothyroxine sodium (LT4) group and wheat grain moxibustion group (WGM) by random number method, with 10 animals in each group, half of which were males and half of which were females; the remaining 20 HT model rats were subjected to experiment II. In order to prevent the natural resilience of rats from leading to a return to normal in models with successful modelling, the same modelling dose of PTU was administered by gavage in subsequent experiments at a frequency of once every other day.

All operations during the experiment were in accordance with the ethics and requirements of animal experimentation, and the study was approved by the Ethics Committee of Shanxi University of Traditional Chinese Medicine (Ethics Approval Number: AWE202409416).

### 2.2 Acupoint selection and dosage of millet moxibustion

According to traditional Chinese medicine (TCM) theory and combined with clinical practice experience, when treating hypothyroidism, acupoints that have the effects of warming and tonifying the spleen and kidneys, as well as assisting yang and benefiting qi, are often selected. The specific acupoints selected include: ‘Dazhui’ (GV14), ‘Mingmen’ (GV4) on the Governor Vessel, bilateral ‘Shenshu’ (BL23) and ‘Pishu’ (BL20) on the Bladder Meridian of Foot-Sun. These acupoints are all located on the back of the body, not only conforming to the principles of TCM syndrome differentiation and acupoint selection but also having good clinical operability.

When selecting the dosage for wheat-grain moxibustion, this experiment referred to clinical experience and conducted preliminary experiments. The results of the preliminary experiments indicated that a low dose (such as 3 moxa cones per acupoint) was less effective; whereas, a high dose (such as 7 moxa cones per acupoint), although potentially effective in the short term, could easily burn the rats’ skin over long-term application. This not only might interfere with the detection of cytokine levels within their bodies but also raises animal ethics issues. Therefore, it was ultimately determined that 5 moxa cones per acupoint is an appropriate dosage, ensuring therapeutic efficacy while avoiding adverse reactions such as skin burns, and meeting ethical requirements for the experiment.

### 2.3 Methods of intervention

**Figure 1:**
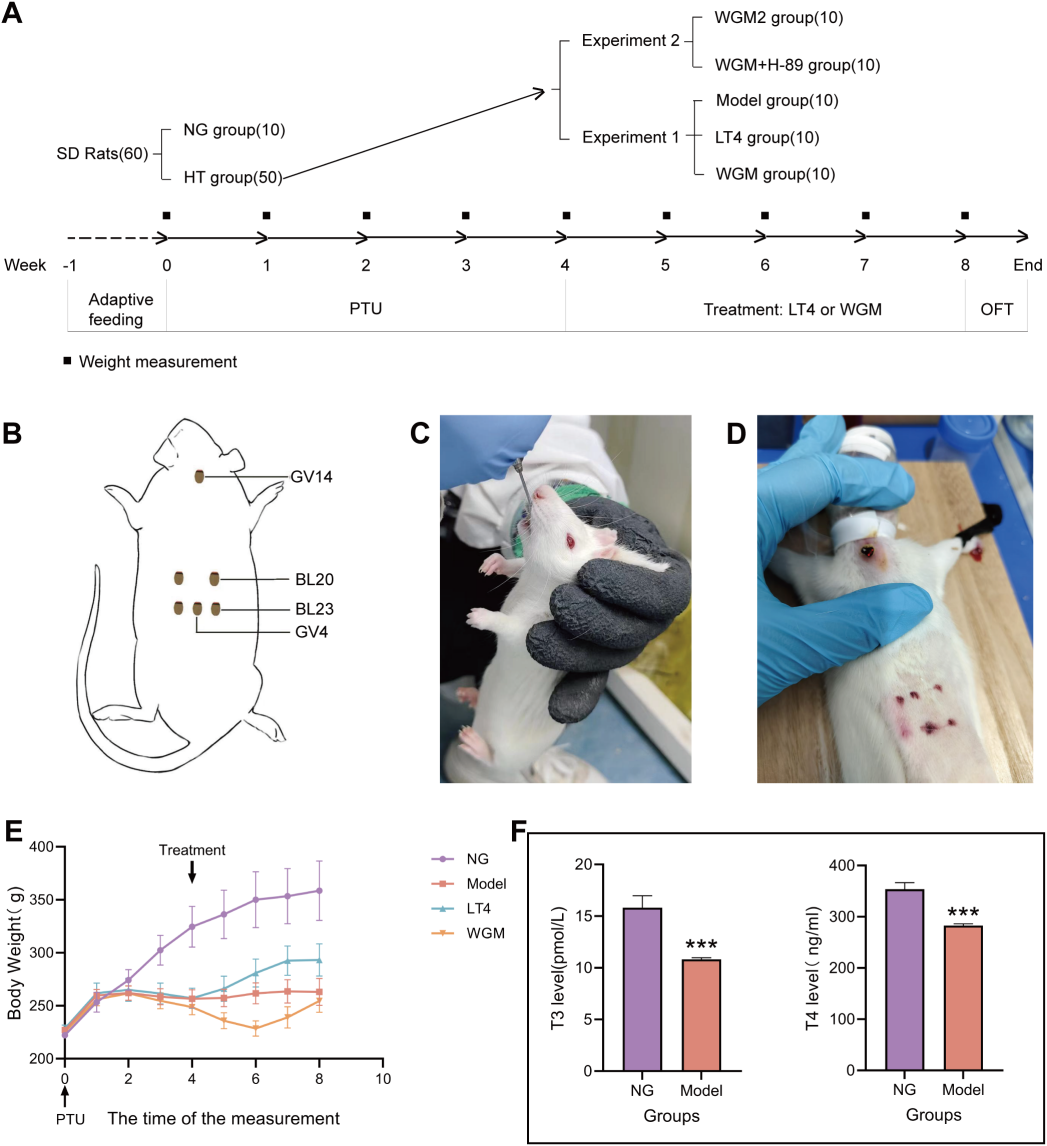
Comparative plots of experimental protocols, body weight measurements and serum T3 and T4 levels in rats after modelling. **(A)** Flow chart of the experiment. **(B)** Schematic diagram of wheat grain moxibustion and acupoint locations. **(C)** Diagram of rats undergoing gavage. **(D)** Detailed diagram of wheat grain moxibustion. **(E)** Graph of weight change of rats. **(F)** Plot of T3 and T4 levels in serum of normal (n=6) and model (n=30) rats after modelling. Results are mean ± standard error, *P<0.05, **P<0.01, ***P<0.001 compared with NG group.

The intervention began one day after completion of the modeling evaluation and continued for four consecutive weeks. Before performing WGM, several preparations were made. First, moxa floss was formed into small pellets, each roughly the size of a wheat grain (approximately 5 mg). Second, six acupoints were identified and marked: “Great Vertebra” (GV14), “Gate of Destiny” (GV4), bilateral “Kidney Shu” (BL23) and bilateral “Spleen Shu” (BL20) (**Figure 1B**). Third, rats were secured in a prone position on a restraining board during moxibustion treatment. The fur over the acupuncture points and the surrounding area (approximately 2 cm in diameter) was shaved. After accurately locating the points, moxa cones were fixed to the skin using Vaseline. The cones were ignited with incense sticks. When approximately three-quarters of each cone had burned, the remaining moxa was removed. Each acupoint received five cones of moxibustion, administered six days per week (**Figure 1D**).

In the LT4 group, a LT4 solution was prepared by dissolving 50 μg of LT4 (batch number: C10011865, Merck Pharmaceutical [Jiangsu]) in 50 mL of physiological saline. Each rat in the LT4 group was then gavaged once daily with 0.9 mL of this solution per 100 g of body weight for four weeks (**Figure 1C**). Except for the NG, all remaining groups received 0.1% PTU solution at a dose of 1 mL per 100 g body weight every other day throughout the experiment. Meanwhile, the blank group was gavaged with an equivalent volume of saline under the same schedule.

### 2.4 Observations on general conditions, body weight, and thyroid tissue

Body weight was measured at a fixed time each week during both the 4-week modeling period and the 4-week treatment period, as well as on the day after treatment concluded (**Figure 1A**), resulting in a total of nine data points per rat. When handling the rats for daily gavage or WGM, observations were made of their general condition, including resistance to handling, tail temperature, the color of the ears, nose, and paw nails. And observe the thyroid tissue as it is taken.

### 2.5 Open field test

The OFT is a classical behavioral paradigm designed to assess spontaneous locomotor activity and exploratory behavior in rodents within a novel environment. This test is primarily employed to evaluate cognitive function, anxiety-like behavior, and exploratory motivation, which collectively reflect the excitability of the central nervous system (CNS). Prior to testing, rats were acclimatized to the experimental room for 1 hour. Throughout the experiment, ambient noise was minimized to maintain a quiet testing environment. Each rat was individually placed in the open field arena, and its behavior was recorded for 3 minutes using a video-tracking system programmed for OFT analysis. The following parameters were quantified: total distance moved, rest time, movement velocity, and frequency of rearing episodes (vertical activity). Heatmap visualizations were generated to represent spatial activity patterns. After each trial, feces and urine were removed, and the arena was thoroughly cleaned with 75% ethanol to eliminate olfactory cues.

### 2.6 Methods of collection

Following the OFT at the end of the treatment period, rats were anesthetized via inhalation of isoflurane (Veterinary Medicine Character 031217015, Hebei Jindafu Pharmaceutical Co.), using 2% for induction and 1.5% for maintenance, delivered in a mixture of 80% N_2_O and 20% O_2_. Blood samples were subsequently collected from the abdominal aorta. The thyroid gland was then carefully excised with a portion of the trachea retained to prevent loss of the small thyroid tissue. Immediately after dissection, tissue samples were visually inspected and divided into two parts. One segment was fixed in paraformaldehyde for subsequent hematoxylin and eosin (H&E) staining; the other was flash-frozen at −80°C for WB and RT-PCR analyses.

### 2.7 Hematoxylin and eosin staining

To perform H&E staining, fixed tissues were removed for ethanol gradient dehydration, embedded using paraffin immersion, and cut into 5 μm size. They were placed on slides, dried and dewaxed, followed by hematoxylin staining, then differentiation, reblue, eosin staining, dehydration, and sealing steps, and observed in 100× and 400× fields of view.

### 2.8 Enzyme-linked immunosorbent assay

After blood sampling from the abdominal aorta, the blood was allowed to stand at room temperature for 2 hours, then centrifuged at 3000 rpm for 10 minutes. The upper layer of serum was collected and stored in a -80°C refrigerator. For ELISA analysis, first retrieve the samples to be tested and thaw them. According to the kit instructions, add the standards and samples to the enzyme-linked immunosorbent assay plates. After adding the enzyme, incubate at 37°C for 60 minutes. Wash five times, then sequentially add the substrate and stop solution. Measure the absorbance at a wavelength of 450 nm using a microplate reader (CMax Plus, Molekula Instruments Co., Ltd). Finally, calculate the content of each indicator based on the standard curve. This experimental method detects serum levels of Triiodothyronine (T3) (ml545079V, mlbio), Thyroxine (T4) (ml102852V, mlbio), TSH (ml002877V, mlbio), Free Triiodothyronine (FT3) (ml233481V, mlbio), FT4 (ml002849V, mlbio), ROS (ml926281, mlbio), MDA (ml059387, mlbio), CAT (ml037079, mlbio), SOD (ml059387, mlbio), GPx (ml077381, mlbio), cAMP (JL10117, Jianglai Biological), a total of 10 indicators.

### 2.9 Western blot analysis

For WB analysis, tissue samples (10 mg) were homogenized to extract total protein. Approximately 30 μg of protein from each sample was mixed with 5× loading buffer, boiled, and centrifuged. The proteins were separated by SDS-PAGE and subsequently transferred onto a PVDF membrane via electroblotting. The membrane was blocked with 5% non-fat milk at room temperature for 1 hour, followed by incubation with primary antibodies at 4°C overnight. The primary antibodies included PKA (27398-1-AP, Proteintech; 1:1000), CREB (12208-1-AP, Proteintech; 1:2000), p-CREB (ab32096, Abcam; 1:500), and PDE3A (ab99236, Abcam; 1:2000). After washing, the membrane was incubated with an HRP-conjugated secondary antibody for 1 hour at room temperature and washed again. Finally, protein bands were visualized using enhanced chemiluminescence reagent and exposed in a dark room for development. This method enabled the detection of PKA, CREB, p-CREB, and PDE3A expression levels in thyroid tissues.

### 2.10 Quantitative real-time polymerase chain reaction

Total RNA was extracted from frozen thyroid tissues using Trizol reagent. Briefly, the samples were pulverized in liquid nitrogen, followed by lysis in Trizol at room temperature. RNA was purified through a series of steps including centrifugation, vigorous mixing, silica membrane adsorption, and washing. Subsequently, the extracted RNA was reverse transcribed into cDNA using a thermal cycler with programmed temperature steps. The resulting cDNA was amplified via real-time PCR using specific primers for PDE3A, PKA, and CREB (**Table 1**). Amplification products were electrophoresed on a 1% agarose gel for 10 minutes and visualized under a gel imaging system.

**Table 1:** PCR primer information sheet.

Name of target gene	Primer sequence (5’-3’)	Product length (bp)
R-Prkaca-F	CCCACTTACGGCGGATTG	265
R-Prkaca-R	GCTTTGTTGTAGCCTTTGC	
R-CREB1-F	AGTGCCCAGCAACCAAGT	113
R-CREB1-R	GGGAGGACGCCATAACAA	
R-PDE3A-F	GAAGTCACCACCAAACGAG	151
R-PDE3A-R	AAGGTCCATCAGCAGGATT	
R-β-actin-F	CTGGCTCCTAGCACCATGAA	180
R-β-actin-R	AAAACGCAGCTCAGTAACAGTC	

### 2.11 Statistical analyses

All data were analyzed and visualized using GraphPad Prism 10.2.3 (GraphPad Software, USA). Significance of differences among groups was evaluated by one-way analysis of variance (ANOVA) or independent-sample t-tests. Results are presented as mean ± standard error, and statistical significance was defined at P < 0.05. Additional significance thresholds were noted at *P* < 0.01 and *P* < 0.001.

## 3 Results

### 3.1 Evaluation of modelling results

After the modeling process was completed, to evaluate the manufacturing effect of the HT model, by observing the general condition of the rats, it was found that the NG group rats had good mental states, were active and flexible, had shiny fur, and their eyes, ears, and noses were red and lustrous, with sensitive reactions to the outside world, and resisted strongly when caught; the rats in the NG group showed steady growth in body weight. Compared to the NG group, the HT model rats exhibited reduced activity, often huddled and curled up, preferred to arch their backs, had cold tails, dull eye, ear, and nose coloration, rough fur, loose skin when handled, and weak resistance. Additionally, the body weight of the hypothyroid model rats increased to a certain point and then ceased to grow (**Figure 1E**). 5 rats were kept in a cage, 3 rats were randomly selected from each cage during the testing of modelling, indicating that there were 6 rats in the NG group, and 30 rats in the HT model were tested for serum levels of T3 and T4 (**Figure 1F**), and found that compared with the NG group, the T3 content of the HT model was significantly down-regulated (*P* < 0.001), the T4 content was significantly down-regulated (*P* < 0.001). The above results indicate that the HT model was induced successfully.

### 3.2 WGM improves the general condition of HT model rats

After four weeks of treatment, the general condition of the NG and Model groups remained consistent with the previous description. Compared to the Model group, rats in both the LT4 and WGM groups demonstrated improved mobility, elevated tail temperature, brighter coloration of the eyes, ears, and nose, as well as stronger resistance during capture. Body weight in the Model, LT4, and WGM groups was significantly lower than that in the NG group, with notable differences and distinct growth curves. Relative to the Model group, the LT4 group exhibited a transition from steady weight gain to a plateau, whereas the WGM group showed a shift from weight loss to a consistent upward trend.

### 3.3 WGM improves behavioural performance in HT model rats

**Figure 2:**
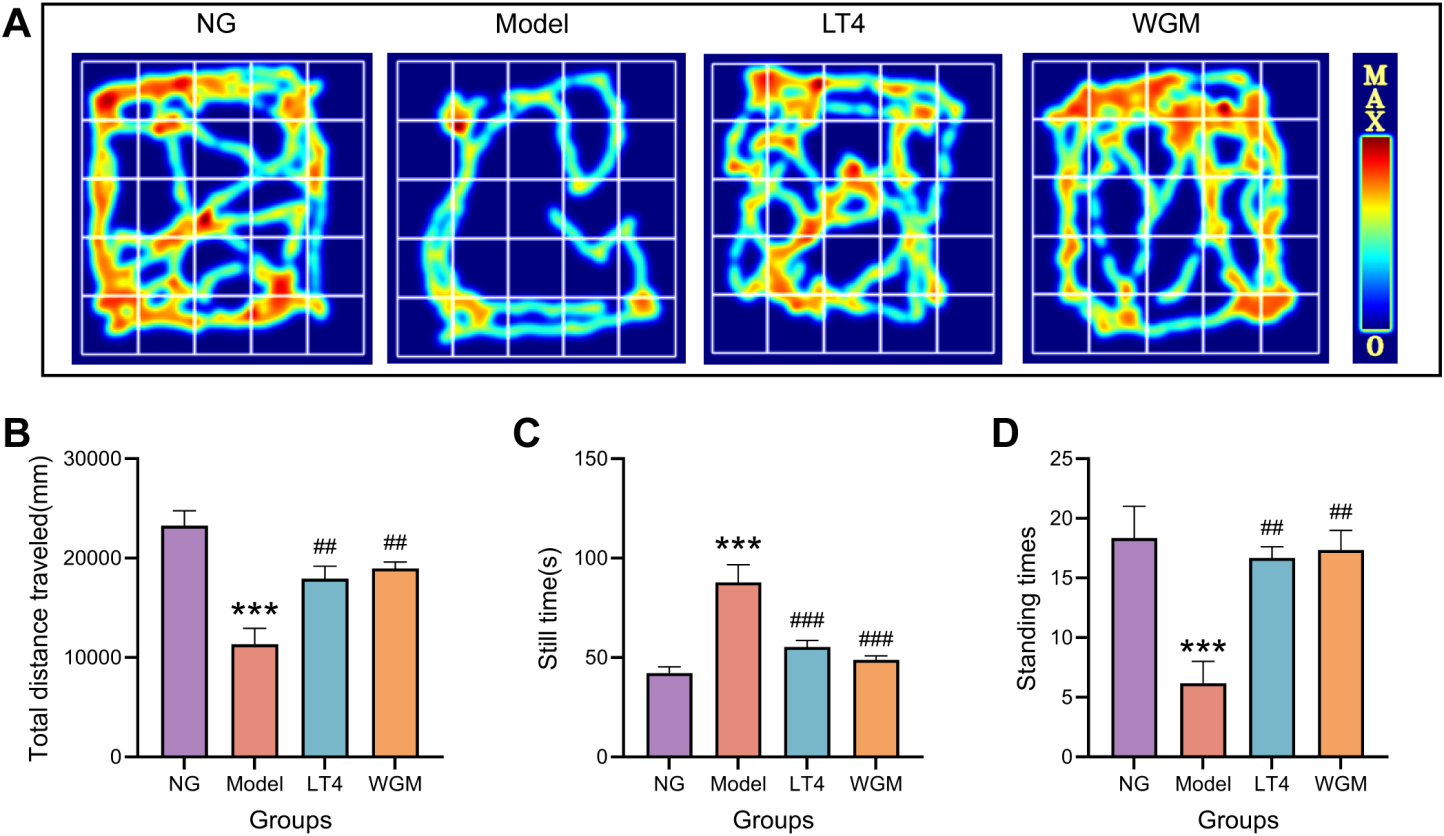
WGM improves OFT performance in HT model rats. **(A)** OFT hotspot map. **(B–D)** Plot of total distance travelled, resting time versus number of vertical stands for each group of rats in the OFT experiment (n=6). Results are mean ± standard error. Compared with the NG group, *P<0.05, **P<0.01, and ***P<0.001; compared with the Model group, *^#^P<*0.05, *^##^P<*0.01, and *^###^P* < 0.001.

To test the strength of the rats’ activity, the OFT test was conducted in this experiment, analyzing the total distance traveled, stationary time, and number of vertical movements (**Figure 2**) with the following findings: Compared with the NG group, the total movement distance was significantly shorter (*P* < 0.001), the resting time was significantly longer (*P* < 0.001), and the number of vertical movements was significantly lower (*P* < 0.001) in the Model group, indicating that the HT rats were less active. Compared with the Model group, both the LT4 group and the WGM group resulted in significantly longer total movement distance (LT4: *P* < 0.01; WGM: *P* < 0.01), significantly shorter resting time (LT4: *P* < 0.001; WGM: *P* < 0.001), and significantly higher number of vertical locomotor movements (LT4: *P* < 0.01; WGM: *P* < 0.01), indicating that both LT4 and WGM could both improve the behavioural performance of HT rats. There was no statistical significance between the indicators in the LT4 and WGM groups, suggesting that the effect of WGM in improving the behavioural performance of HT rats did not differ or was not significantly different from that of LT4.

### 3.4 WGM improves the destruction of thyroid tissue structure and enlargement of thyroid tissue in HT model rats

**Figure 3:**
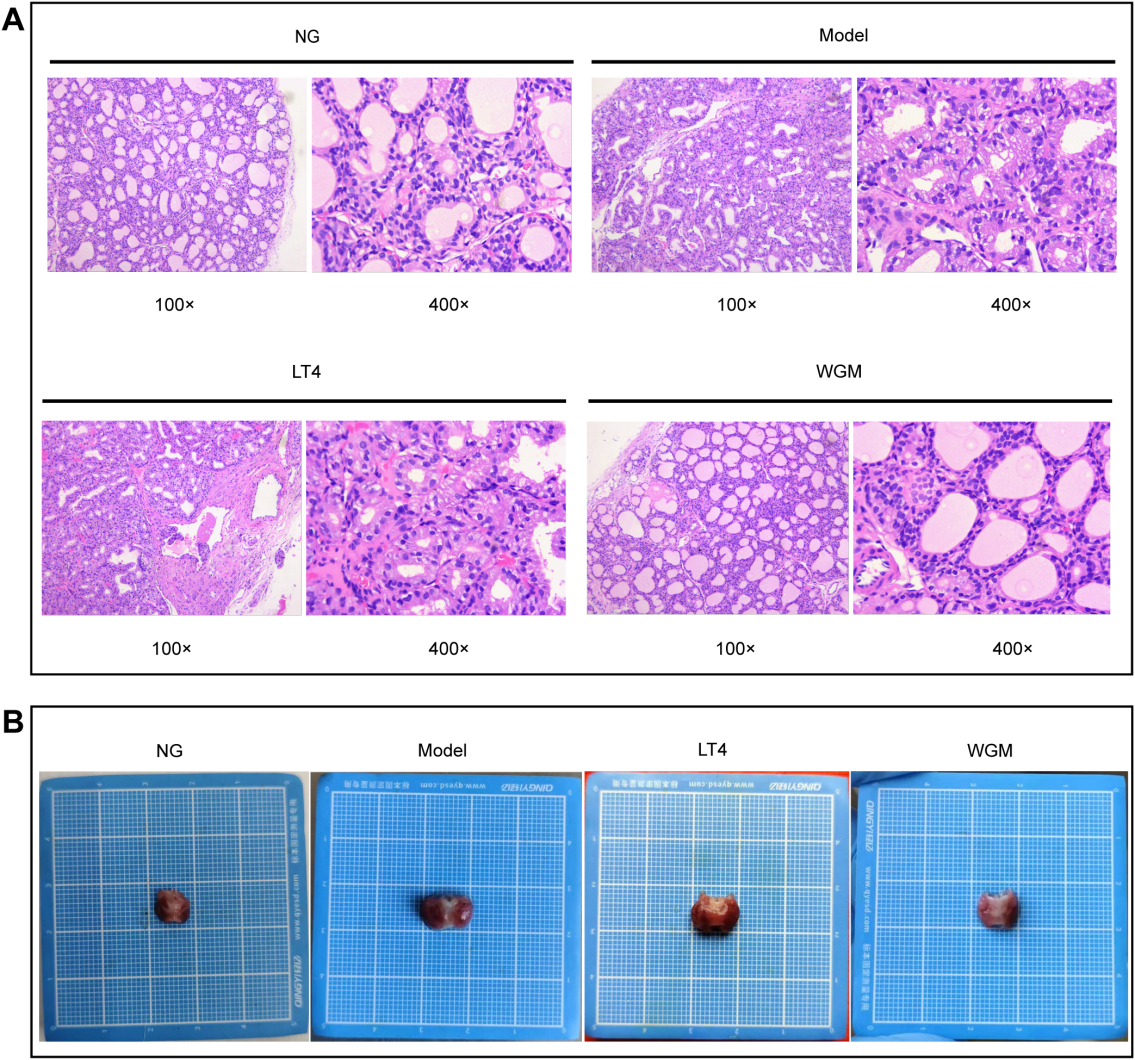
WGM improves thyroid tissue structure in HT model rats. **(A)** H&E-stained histogram of the thyroid gland (n=3). **(B)** Histogram of the thyroid gland.

To clearly define structural changes in thyroid tissue during treatment, histomorphology is assessed using H&E staining (**Figure 3A**). In the NG group, follicles exhibited uniform size, lined by cuboidal to flattened epithelial cells, with lumina filled with colloid. The model group showed obliteration of follicular lumina due to epithelial hyperplasia. Epithelial cells adopted a tall columnar or papillary morphology, projecting into the lumen. Follicles displayed considerable size variation and a marked reduction in colloid content. In the LT4 group, follicular lumina were effaced by hyperplastic epithelium consisting of cuboidal to tall columnar cells. Follicles in the WGM group appeared uniformly sized, composed of cuboidal or flattened epithelial cells, and contained colloid-filled lumina. Histological analysis revealed that HT induced structural disruption of thyroid tissue, characterized by a shift in follicle morphology from uniformly sized round or oval shapes to irregularly sized and flattened forms. This alteration was accompanied by changes in epithelial cell phenotype, along with a reduction in colloid content. LT4 treatment failed to restore the thyroid architecture. In contrast, WGM effectively reversed follicular disruption, normalized epithelial cell morphology, and increased colloid deposition.

After sampling, the thyroid tissue was observed (**Figure 3B**). Compared with the NG group, thyroid tissue exhibited varying degrees of enlargement in the Model, LT4, and WGM groups. The most pronounced enlargement was observed in the Model group, followed by the LT4 group, while the WGM group displayed the mildest change.

### 3.5 WGM improves FT3, FT4 and TSH levels in serum of HT model rats

**Figure 4:**
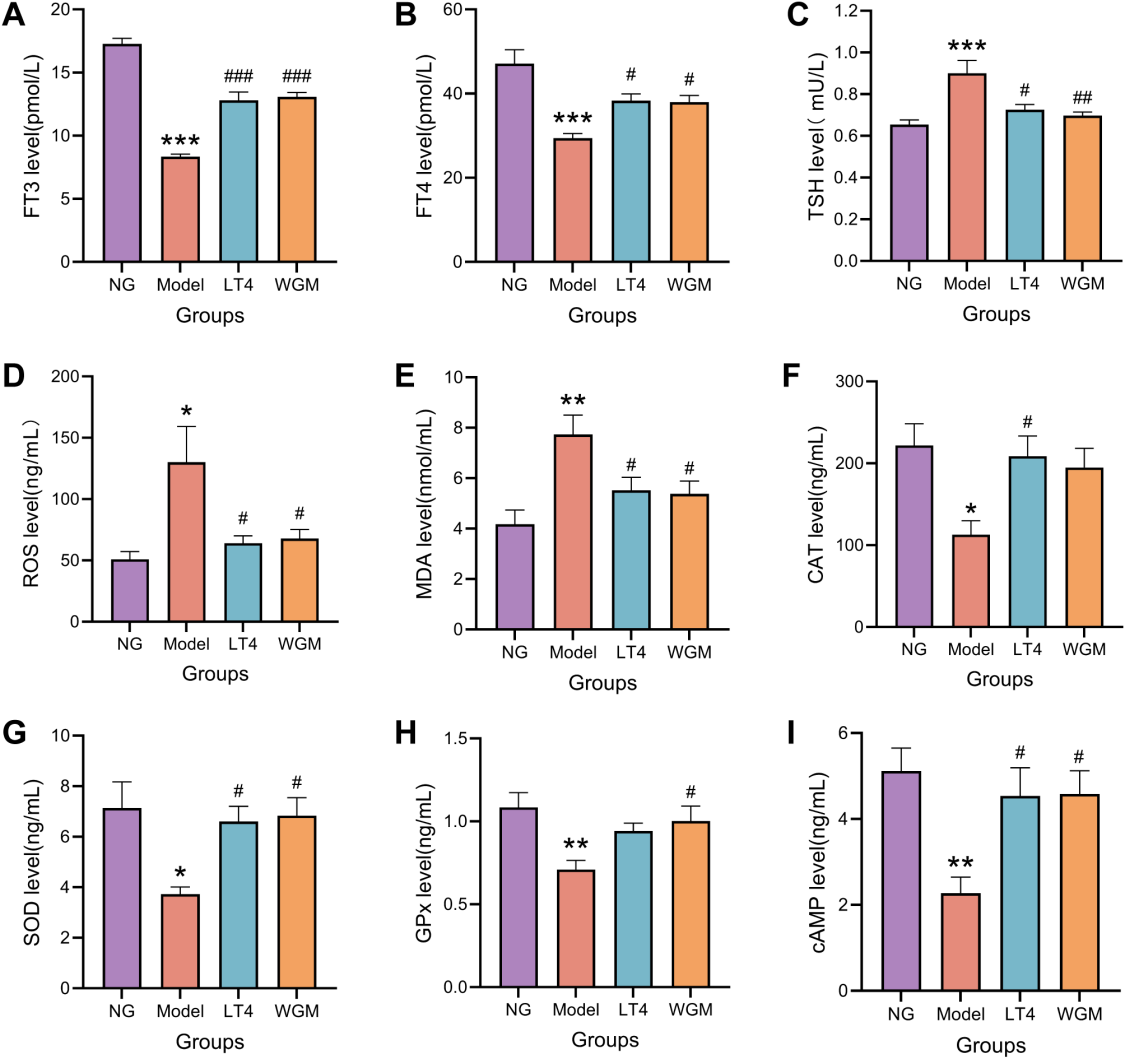
WGM improves thyroid hormone and cAMP content and oxidative stress status in HT model rats. **(A–C)** Comparison of serum levels of FT3, FT4, and TSH in rats from each group (n=6). **(D–H)** Comparative plots of serum levels of ROS, MDA, CAT, SOD, and GPx in rats in each group (n=6). **(I)** Comparative plots of cAMP levels in serum of rats in each group (n=6). Results are mean ± standard error. Compared with the NG group, *P<0.05, **P<0.01, ***P<0.001; compared with the Model group, *^#^P* < 0.05, *^##^P* < 0.01, *^###^P* < 0.001.

To assess the effect of WGM on HT model rats, serum levels of FT3, FT4, and TSH were measured using ELISA (**Figures 4A–C**). Compared with the NG group, the Model group exhibited significantly reduced FT3 (*P* < 0.001) and FT4 levels (*P* < 0.001), along with a marked increase in TSH (*P* < 0.001), confirming the successful maintenance of the HT model throughout the experimental period. Relative to the Model group, both the LT4 and WGM groups showed significant upregulation of FT3 (LT4: *P* < 0.001; WGM: *P* < 0.001), upregulation of FT4 (LT4: *P* < 0.05; WGM: *P* < 0.05), and downregulation of TSH (LT4: *P* < 0.05; WGM: *P* < 0.01). These results indicate that both interventions effectively reversed the HT-induced alterations in thyroid hormone levels. No statistically significant differences were observed between the WGM and LT4 groups, implying comparable efficacy in modulating thyroid hormone content.

### 3.6 WGM improves serum levels of ROS, MDA, CAT, SOD & GPx in HT model rats

To clarify the regulatory effect of WGM on the imbalance of oxidation and antioxidation in HT model rats, ELISA was used to detect the levels of ROS, MDA, SOD, and GPx in serum (**Figures 4D–H**), with the results as follows. Compared with the NG group, the ROS content was up-regulated (*P* < 0.05), the MDA content was significantly up-regulated (*P* < 0.01), the CAT content was significantly down-regulated (*P* < 0.001), the SOD content was down-regulated (*P* < 0.05), and the GPx content was significantly down-regulated (*P* < 0.01) of the Model group, which suggests that HT causes an up-regulation of oxidative capacity and a down-regulation of antioxidative capacity in rats, resulting in OS, destroying the cell structure, and then harming the functions of various organs. Compared with the Model group, the LT4 group and the WGM group exhibited down-regulation of ROS content (LT4: *P* < 0.05; WGM: *P* < 0.05), down-regulation of MDA content (LT4: *P* < 0.05; WGM: *P* < 0.05), a significant up-regulation of CAT content (LT4: *P* < 0.01; WGM: *P* < 0.01), an up-regulation of SOD content (LT4: *P* < 0.05; WGM: *P* < 0.05), and GPx content was similarly upregulated (WGM: *P* < 0.05), but the difference in the LT4 group was not statistically significant. There was no statistically significant difference in the content of the indicators between the LT4 group and the WGM group, suggesting that WGM can improve the OS of HT, and that its effect is no or very little different from that of LT4.

### 3.7 WGM improves cAMP levels in serum of HT model rats

To investigate whether the cAMP signaling pathway plays a key role in the mechanism of action of wheat-grain moxibustion, the content of cAMP in the serum was first detected using the ELISA method (**Figure 4I**). Compared with the NG group, cAMP content was significantly down-regulated in the Model group (*P* < 0.01); compared with cAMP content in the Model group, it was up-regulated in the LT4 group (*P* < 0.05) and up-regulated in the WGM group (*P* < 0.05); the difference between cAMP content in the LT4 group and the WGM group was not statistically significant. The above results suggest that HT decreases cAMP content, but LT4 and WGM reverses the decrease in cAMP.

### 3.8 WGM improves PKA, p-CREB/CREB, and PDE3A protein expression in thyroid tissues of HT model rats

**Figure 5:**
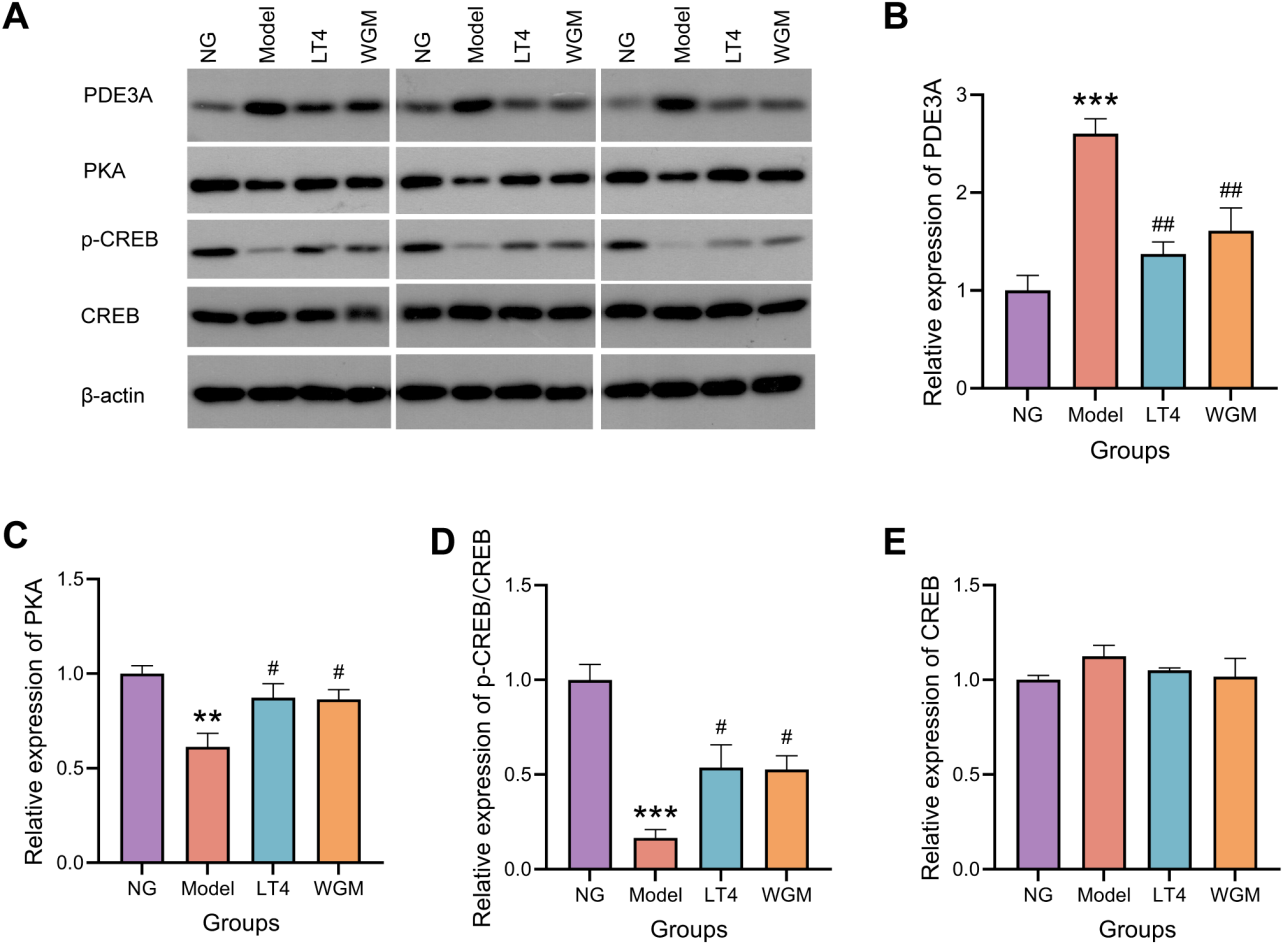
WGM improves cAMP pathway protein expression in thyroid tissue. **(A)** WB bands. **(B)** PDE3A protein expression in thyroid tissue (n=3). **(C)** PKA protein expression in thyroid tissue (n=3). **(D)** p-CREB protein expression in thyroid tissue (n=3). **(E)** CREB protein expression in thyroid tissue (n=3). Results are mean ± standard error. Compared with the NG group, *P<0.05, **P<0.01, ***P<0.001; compared with the Model group, *^#^P* < 0.05, *^##^P* < 0.01.

To further investigate the role of the cAMP signaling pathway, the protein expression of downstream molecules PKA, CREB, p-CREB, and the cAMP degradation molecule PDE3A was detected using WB method (**Figure 5**). Compared with the NG group, PKA expression was significantly down-regulated (*P* < 0.01), CREB expression was essentially unchanged, p-CREB/CREB was significantly down-regulated (*P* < 0.001), and PDE3A expression was significantly up-regulated (*P* < 0.001) in the Model group, which suggests that HT causes up-regulation of PDE3A expression, resulting in down-regulation of cAMP content, down-regulation of its downstream molecule PKA expression, and down-regulation of CREB phosphorylation levels. Compared with the Model group, both the LT4 group and the WGM group showed a significant down-regulation of PDE3A expression (LT4: *P* < 0.05; WGM: *P* < 0.01), an up-regulation of PKA expression (LT4: *P* < 0.05; WGM: *P* < 0.05), and an up-regulation of p-CREB/CREB (LT4: *P* < 0.05; WGM: *P* < 0.05), CREB expression was shown to be essentially flat. There was no statistically significant difference in the expression of the indicators between the LT4 group and the WGM group. LT4 and WGM decreased PDE3A expression, increased cAMP content and PKA expression, and upregulated CREB phosphorylation levels, reversing the inhibition of the signalling pathway caused by HT. This suggests that the OS situation was improved and the cAMP signalling pathway played a role in the process of WGM.

### 3.9 WGM improves PDE3A and PKA mRNA expression in thyroid tissues of HT model rats

**Figure 6:**
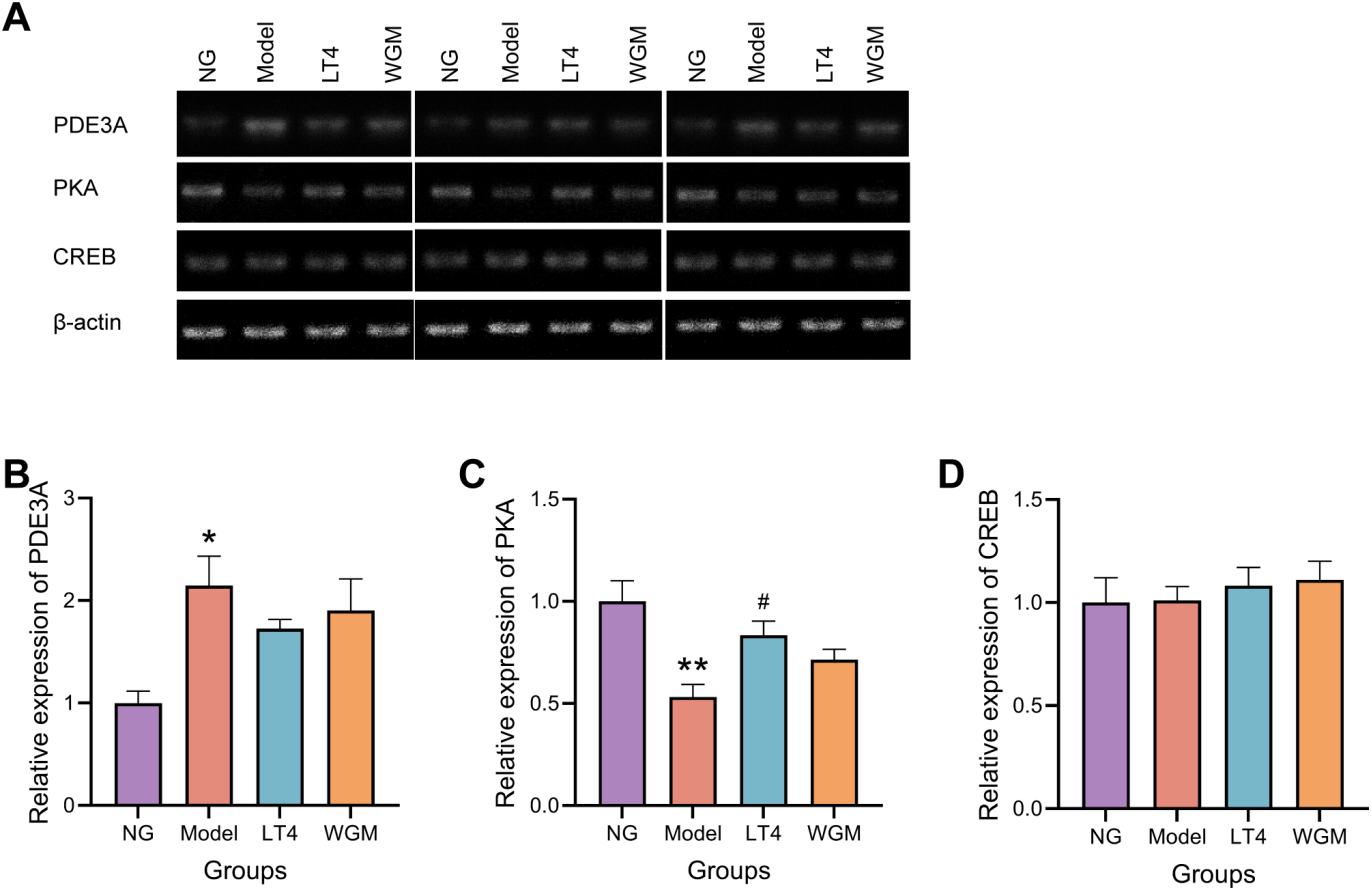
WGM improves cAMP pathway protein mRNA expression in thyroid tissue. **(A)** RT-PCR bands. **(B)** PDE3A mRNA expression in thyroid tissue (n=3). **(C)** PKA mRNA expression in thyroid tissue (n=3). **(D)** CREB mRNA expression in thyroid tissue (n=3). Results are mean ± standard error. Compared with the NG group, *P<0.05, **P<0.01, ***P<0.001; compared with the Model group, *^#^P* < 0.05, *^##^P* < 0.01.

Compared with the NG group, PDE3A mRNA expression was elevated (*P* < 0.05), PKA mRNA expression was significantly lower (*P* < 0.01), and CREB mRNA expression was not significantly different in the Model group. Compared with the Model group, PDE3A mRNA expression was reduced but not statistically significant, PKA mRNA expression was elevated (*P* < 0.05), and CREB mRNA expression was not significantly different in the LT4 group; Compared with the Model group, there was no statistically significant difference in CREB mRNA expression, although PDE3A mRNA expression was reduced and PKA mRNA expression was elevated in the WGM group (**Figure 6**).

However, whether WGM improves oxidative stress through the cAMP signaling pathway still needs further confirmation. To this end, Experiment II of this study detected and analyzed relevant indicators.

## 4 Experiments II and results

To further elucidate the role of this signaling pathway in the intervention mechanism, the inhibitor H-89 (N857858-250mg, Macklin) was introduced in the experiment II. Twenty successfully modeled SD rats were randomly and evenly assigned to two groups: Wheat-Grain Moxibustion Group 2 (WGM2) and Wheat-Grain Moxibustion plus H-89 Group (WGM+H-89), with 10 rats per group (equal numbers of males and females). The experimental procedures remained consistent with those of the WGM group in prior experiments, except that the WGM+H-89 group received intraperitoneal injections of H-89 solution during the moxibustion treatment. The H-89 solution was prepared by first mixing 2 mL dimethyl sulfoxide (DMSO, Dogesce), 8 mL PEG300 (HARVEYBI), 1 mL Tween-80 (Dogesce), and 9 mL physiological saline to obtain 20 mL of solvent. Then, 20 mg of H-89 powder was dissolved in this solvent to yield the final injection solution. The H-89 solution was administered intraperitoneally at a dosage of 1 mL per 200 g body weight within 30 minutes prior to each WGM session. The experimental outcomes following combined inhibitor and WGM treatment are presented below.

### 4.1 H-89 reduced PKA, p-CREB/CREB protein expression with PKA mRNA expression in thyroid tissue

**Figure 7:**
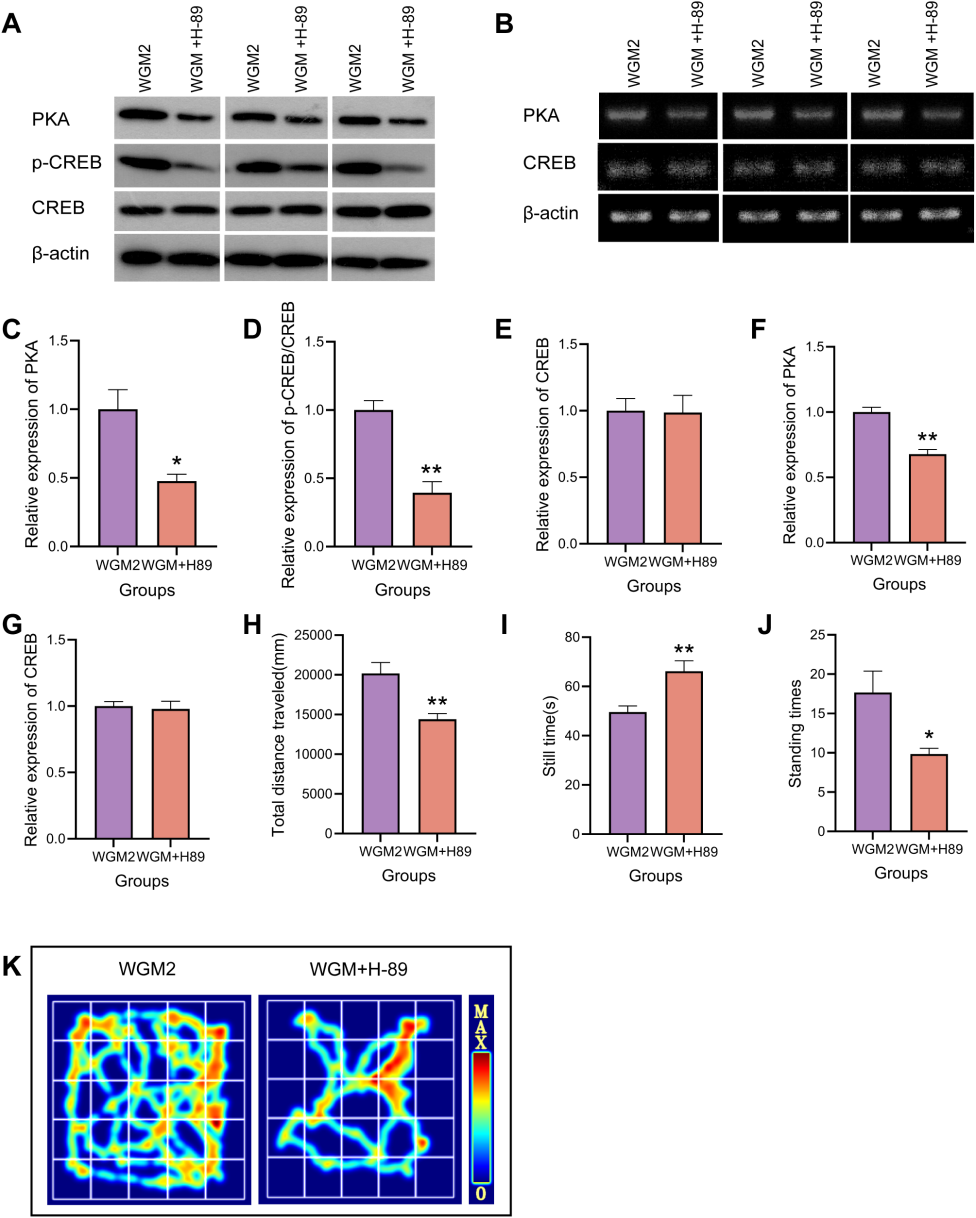
H-89 reverses cAMP pathway protein and mRNA expression and OFT manifestation in thyroid tissue. **(A, C–E)** WB bands and PKA, p-CREB, CREB protein expression in thyroid tissue (n=3). **(B, F, G)** RT-PCR bands and PKA, CREB mRNA expression in thyroid tissue (n=3). **(H–J)** Plot of total distance travelled, resting time versus number of vertical stands for each group of rats in the OFT experiment (n=6). **(K)** OFT hotspot map. Results are mean ± standard error, *P<0.05 and **P<0.01 compared with the WGM2 group.

To verify the inhibitory effect of H-89 on the cAMP signaling pathway, WB was used to detect the protein expression levels of PKA and p-CREB/CREB in thyroid tissue, and RT-PCR was used to measure the mRNA expression level of PKA in thyroid tissue (**Figures 7A–G**). Compared with the WGM2 group, PKA expression was reduced (*P* < 0.05), CREB phosphorylation level was significantly down-regulated (*P* < 0.01), PKA mRNA expression was reduced (*P* < 0.01), and there was no significant difference in both CREB protein expression and CREB mRNA expression in the WGM+H-89 group. The above results indicate that the signalling pathway is inhibited.

### 4.2 H-89 reverses the ameliorative effect of WGM on general condition and behavioural studies in HT model rats

Relative to the WGM2 group, the rats in the WGM+H-89 group were sluggish in their activities, curled up in piles, had cool tails, and had light coloured eyes, ears, and noses. In the OFT, they showed a significantly shorter total movement distance (*P* < 0.01), a significantly longer resting time (*P* < 0.01), and a significantly lower number of vertical movements (*P* < 0.05). These results suggests that H-89 reversed the ameliorative effect of WGM on the general condition and behavioural performance of HT model rats, a process related to the cAMP/PKA signalling pathway (**Figures 7H–K**).

### 4.3 H-89 reverses the improvement of thyroid histological structure in HT model rats by WGM

**Figure 8:**
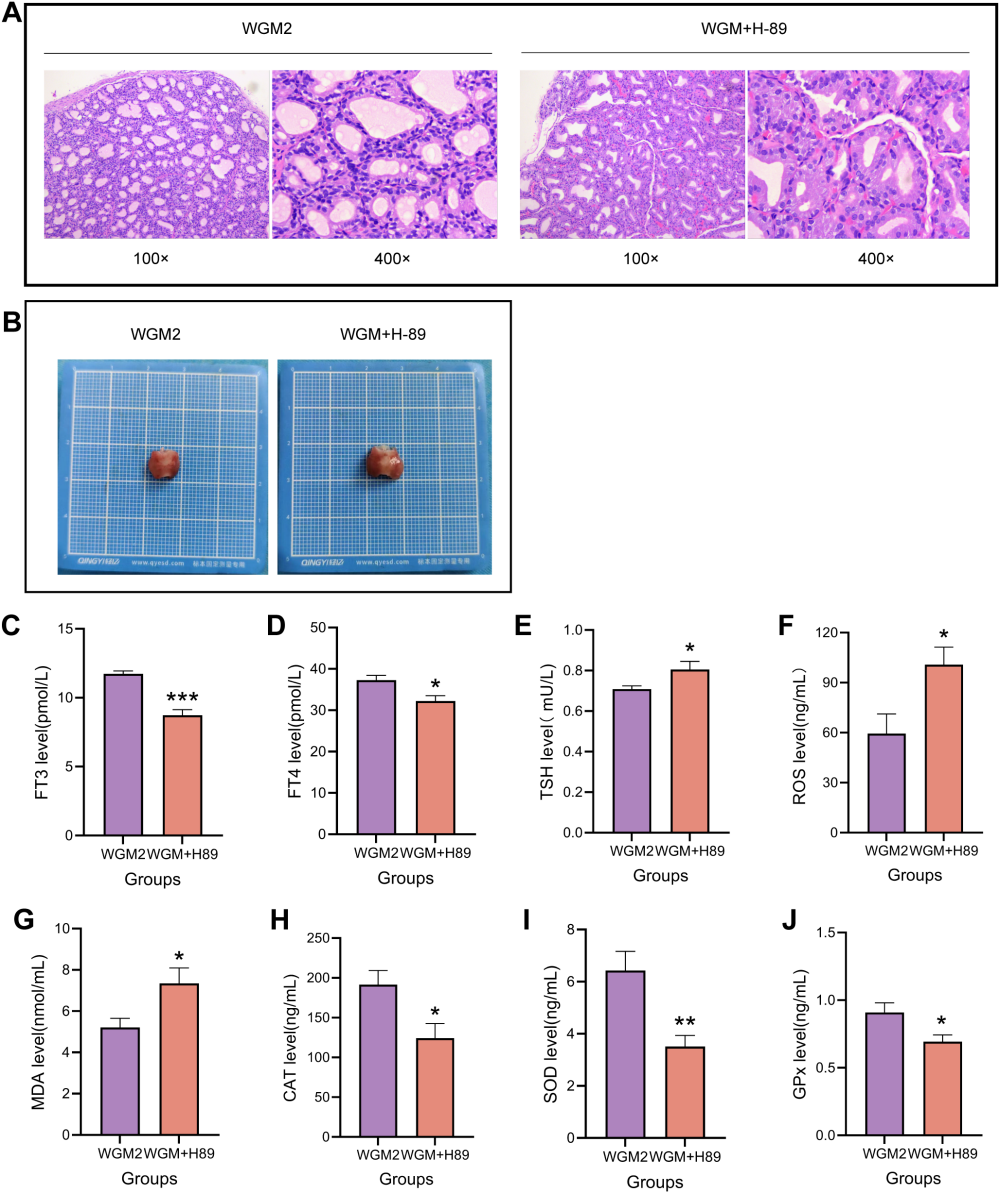
H-89 reversed thyroid tissue structure with serum thyroid hormones and oxidative stress. **(A)** H&E-stained histogram of the thyroid gland (n=3). **(B)** Histogram of the thyroid gland. **(C–E)** Comparison of serum levels of FT3, FT4, and TSH in rats in each group (n=6). **(F–J)** Comparative plots of serum levels of ROS, MDA, CAT, SOD, and GPx in rats in each group (n=6). Results are mean ± standard error, *P<0.05, **P<0.01, ***P<0.001 compared with the WGM2 group.

WGM2 group: follicles were of uniform size, the epithelial cells were cuboidal or flattened, and the follicular lumen was filled with colloid; WGM+H-89 group: the epithelial cells showed hypercolumnar or papillary hyperplasia, the follicular lumens were of uneven size, and the colloid content was reduced. H-89 resulted in disruption of the improvement of thyroid histological structure by WGM in HT model rats and relative enlargement of the thyroid gland (**Figures 8A, B**).

### 4.4 H-89 reversed the ameliorative effect of WGM on serum levels of FT3, FT4 and TSH in HT model rats

Compared with the WGM2 group, the FT3 content was significantly down-regulated (*P* < 0.001), the FT4 content was down-regulated (*P* < 0.05) and the TSH content was up-regulated (*P* < 0.05) in the WGM+H-89 group. It indicates that after inhibition of the signalling pathway, the ameliorative effect of WGM on thyroid hormones in HT rats was reduced (**Figures 8C–E**).

### 4.5 H-89 reversed the ameliorative effect of WGM on serum levels of ROS, MDA, CAT, SOD and GPx in HT model rats

Compared with the WGM2 group, ROS content was up-regulated (*P* < 0.05), MDA content was up-regulated (*P* < 0.05), CAT content was significantly down-regulated (*P* < 0.001), SOD content was significantly down-regulated (*P* < 0.01), and GPx content was down-regulated (*P* < 0.05) in the WGM+H-89 group. These manifestations suggest that when the cAMP signalling pathway was inhibited, the ameliorative effect of WGM on oxidative stress in HT rats was reversed (**Figures 8F–J**).

## 5 Discussion

Moxibustion therapy has a long history in China as a method of treatment. Moxibustion activates local acupoints, thereby inducing changes in blood composition and hemodynamics. It regulates vascular dilation and constriction, as well as the release of inflammatory factors. Furthermore, moxibustion modulates the neuro-endocrine-immune network, ultimately promoting the recovery of visceral functions. It is considered that the main biological mechanism of moxibustion’s heat radiation effect is its warming and unblocking properties (26). Modern studies have shown that moxibustion enhances steroidogenesis in granulosa cells by activating the cAMP/PKA/CREB pathway (27), improves ovarian function impairment, and enhances the function of the amygdala and hypothalamic-pituitary-adrenal axis in rats. It also has advantages in regulating abnormal reproductive hormones (28) and plays an important role in the treatment of endocrine system diseases. Moxibustion can inhibit excessive autophagy, regulate the expression of immune-related factors, reduce inflammation, and has advantages in regulating immune function (29). Ancient texts mention various moxibustion methods for goiter, such as moxibustion on the Dazhui point to treat goiter, indicating that the use of moxibustion for treating goiter has a long history and that there are specific acupoints chosen for moxibustion.

Thyroid hormones have a wide range of roles and play an important role in human growth, reproduction, neuronal development and regulation of energy metabolism. The hypothalamic-pituitary-thyroid axis regulates thyroid hormone secretion. TSH, produced and released by the pituitary gland, stimulates both the synthesis and secretion of thyroid hormones (30). HT is an endocrine disorder resulting from impaired synthesis and secretion of thyroid hormones. This condition downregulates the body’s metabolic rate, potentially leading to severe and life-threatening multi-system complications involving the neurological, endocrine, digestive, and cardiovascular systems (31). After confirming HT, it can be found that indicators reflecting the synthesis and secretion status of thyroid hormones will change accordingly, such as an appropriate increase in serum TSH levels, while serum FT4, T3, and T4 levels decrease (32). The use of PTU gavage to induce the model of HT is a well-established method and is an important modelling modality when conducting HT research (33, 34), which is consistently used by the subject group. At the end of the 4-week modeling period using PTU, serum levels of T3 and T4 were measured in rats. Subsequently, TSH, FT4, and FT3 levels were detected in both the NG group and model group after 4 weeks of treatment. The results were consistent with HT characteristics, indicating successful establishment of the HT model. Following WGM treatment, serum FT4 and FT3 levels were up-regulated, while TSH levels were down-regulated in HT rats, suggesting that WGM can ameliorate HT by modulating thyroid hormone homeostasis.

When thyroid hormone is deficient, the metabolic rate is downregulated and patients typically exhibit cold intolerance, weight gain, constipation, fatigue, hair loss, dry skin, and recoverable cognitive impairment (35). HT rats show signs of bunching and lethargy, lighter colouring of eyes, ears and nose, dulling of external stimuli and reduced resistance to capture. Thyroid hormones are closely linked to CNS (36), and when TH is low, neurotransmission is impaired due to downregulation of CNS energy supply caused by downregulation of glucose metabolism (37), which is responsible for the occurrence of a number of brain diseases such as Alzheimer’s, dementia, Parkinson’s disease (38). OFT reflects CNS excitability to some extent, and HT rats show behavioural inhibition in OFT such as shorter total movement distance, longer resting time, and fewer stand-ups (39), after WGM, reversed the manifestation of behavioural inhibition in HT rats.

Structurally, the functional unit of the thyroid gland is the follicle, formed by the association of specialised epithelial cells, which is filled with a gelatinous mass rich in thyroglobulin and glycoproteins (40). After using the PTU induction model, the structure of the thyroid gland was altered, the follicular lumen disappeared to be filled with hyperplastic epithelium, the epithelial cells protruded into the follicular lumen in the form of high columns or mammillary projections, the follicular lumen was of unequal size, and the gelatinous content was reduced, which is in agreement with the results of some studies (41, 42). After WGM, the structural changes in the thyroid tissue were reversed, moving closer to the structural expression of the NG group. LT4 did not reverse the structural changes in the thyroid tissue, which is probably the biggest difference between WGM and levothyroxine tablets.

There are many studies show that many thyroid disorders, including HT, are closely related to OS. HT is associated with reduced antioxidant utilization (43). Persistent stimulation by elevated TSH levels increases H_2_O_2_ production (44), which serves as a substrate for thyroid hormone synthesis. However, impaired TPO activity prevents the effective use of H_2_O_2_ for tyrosine iodination and iodothyronine coupling. Consequently, inadequate antioxidant protection leads to OS (45) and promotes apoptosis (46). OS is characterised by excess ROS levels that damage DNA, proteins or lipids (47). PTU-induced HT rats exhibited increased serum levels of ROS and MDA, accompanied by decreased levels of SOD, CAT, and GPx. These findings indicate an imbalance between oxidative stress and antioxidant capacity, confirming the presence of OS in HT rats. This result is in line with a number of studies showing that HT increases OS and interferes with cellular life and signal transduction (48), generating oxidative stress in multiple organs (49). After WGM, ROS content was down-regulated, MDA content was down-regulated, SOD content was up-regulated, and GPx content was up-regulated, suggesting that imbalance between oxidation and antioxidant may be regulated by wheat moxibustion. And there are studies proving that moxibustion can enhance the body’s antioxidant capacity (50). It suggests that WGM can achieve improvement in HT by modulating OS.

The regulation of OS is closely related to the cAMP/PKA signalling pathway, and it has been shown that activation of the cAMP/PKA pathway through down-regulation of cellular cAMP-specific PDE activity can inhibit oxidative stress and chemotaxis (51, 52). Increased PDE3A content, decreased cAMP content, decreased PKA content, and decreased CREB phosphorylation in HT model rats suggested that the signalling pathway was inhibited, but which was reversed by WGM. Although the inhibition or activation of signaling pathways is frequently linked to the development and alleviation of OS, it remains unclear whether WGM ameliorates OS in HT rats via the cAMP/PKA signaling pathway. To investigate this, Experiment II was conducted by adding the cAMP/PKA signaling pathway inhibitor H-89. The results showed that after adding H-89, the improvement of OS in HT model rats by WGM was reversed, indicating that WGM may improve oxidative stress by downregulating PDE3A, activating the cAMP/PKA signaling pathway, and promoting CREB phosphorylation.

Additionally, TSH binds to the thyrotropin receptor on thyroid follicular epithelial cells, stimulating thyroid growth as well as hormone synthesis and secretion through cAMP-mediated signaling (53). However, studies have revealed that high TSH levels suppress cAMP production, whereas low TSH levels enhance it, through activation of the cAMP/PKA pathway (54). This bidirectional regulation is mediated by Gs proteins at low TSH concentrations and by Gi/Go proteins at high concentrations, which may serve as a protective mechanism against overstimulation of TSH receptor-expressing cells (55). Another suggestion is that the marked activation of the cAMP/PKA pathway by high concentrations of TSH leads to increased proliferation, which may be inhibited by the PLC-PKC pathway via Gq (56), thereby maintaining the balance of endogenous proliferative capacity of thyroid cells, are complex intertwined molecular mechanisms. In this study, cAMP content was found to be downregulated in HT model rats, which may be the result of bidirectional regulation, and after WGM, cAMP content was upregulated and the pathway was activated, which represents the thyroid function in a good direction.

## 6 Limitations

Although this study provides evidence that WGM can affect OS and thereby improve HT in model rats via the cAMP/PKA signalling pathway, it is important to recognise several limitations. First, thyroid hormones exert actions across multiple organ systems; however, this study was confined to analyses of serum and thyroid tissue. Second, this study used a single PTU-induced HT rat model, which may limit the applicability of the findings to other HT models or to humans. Third, for assessing the rats’ behavioural competence, only a single OFT was selected, which could not fully reflect the depth of the relationship between the rats’ behavioural competence and the HT and CNS. Fourth, the administration of fixed doses of LT4 and WGM may not accurately represent dose-response relationships. More comprehensive insights could be obtained through studies incorporating varied dosage regimens. Fifth, the long-term effects and sustainability of the ameliorative effects of WGM on HT model rats were not assessed. Finally, this study did not delve into the potential side effects or safety of WGM, which is critical for therapeutic applications. Subsequent efforts should be made to address these limitations by combining different HT models, different doses of WGM with LT4, advanced and comprehensive biochemical assays, long-term evaluations, and safety assessments, in order to clearly grasp the potential of WGM in improving HT.

## 7 Conclusion

**Figure 9:**
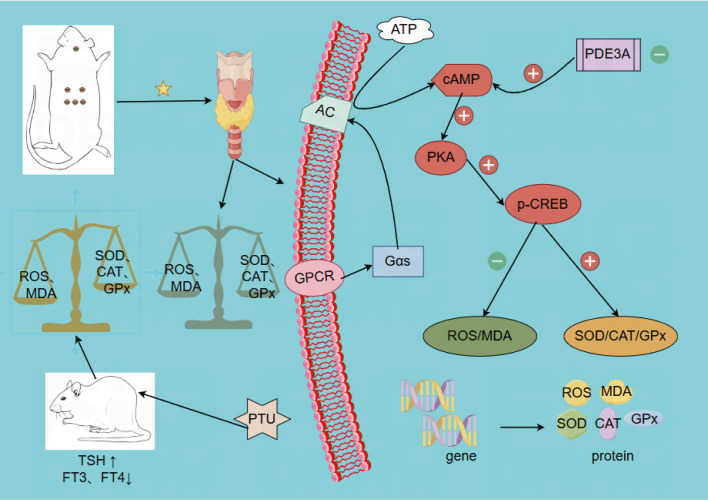
WGM ameliorates oxidative stress in the hypothyroid model rats through activation of the cAMP/PKA signalling pathway.

The present study demonstrated the therapeutic potential of WGM in ameliorating HT in a rat model. WGM administration improved the general condition of HT rats. According to the OFT, WGM restored locomotor activity and enhanced behavioral performance. Histological examination via H&E staining revealed that WGM alleviated structural damage in thyroid tissue. Serological analysis indicated that WGM modulated serum levels of FT3, FT4, and TSH, contributing to the restoration of hormonal homeostasis. Notably, WGM intervention significantly reduced serum levels of ROS and MDA, while increasing the activities of CAT, SOD, and GPx, suggesting a substantial attenuation of oxidative stress. Further mechanistic investigation using the pathway inhibitor H-89 revealed that this effect may be mediated through downregulation of PDE3A, activation of the cAMP/PKA signaling pathway, and promotion of CREB phosphorylation (**Figure 9**). Compared to the LT4-treated group, WGM exhibited superior efficacy in improving thyroid tissue morphology. However, no statistically significant differences were observed between the two groups in terms of oxidative stress mitigation or regulation of associated protein expression. These findings suggest that WGM may serve as a promising therapeutic strategy for HT, warranting further validation in subsequent clinical studies.

## Data Availability

The raw data supporting the conclusions of this article will be made available by the authors, without undue reservation.
